# Annotations

**Published:** 1898-01-01

**Authors:** 


### ANNOTATIONS.

### A Final Touch.

A CASE is reported from San Francisco in which a girl contracted a contagious disease—syphilis, to wit—in a manner which one might consider merely curious did one not know to what an extent people will go in submitting to whatever their barbers and hairdressers choose to do to them. In this case the ulcer was situated on "the left side of the vermilion border of the lower lip." At first great difficulty was experienced in discovering its origin. After a time, however, it came out that the patient had been in the habit of going to a hair-dressing establishment, the *clientèle* of which appeared to be, at the least, somewhat mixed. As she said, "in the shop where she usually went to have her hair dressed she had noticed many 'chemical blondes' and otherwise strikingly dressed women." Now at this shop it seems that a very filthy and dangerous custom prevailed. "The hairdresser, as a final touch, drew a 'rouge stick' across the lips of her customers." This is bad enough—and we wonder whether this little "final touch" is common in London—but after all, it is only one step worse than the dirty little brush with which most barbers try to daub brilliantine on to the moustaches of their more submissive clients. In the account from San Francisco, however, there is worse to follow. "The 'rouge stick' is a cylinder composed of a firm red ointment. The firmness necessitates a slight moistening before being applied, and, disgusting to relate, this is frequently accomplished by the hairdresser first putting it to her own mouth and then deftly drawing it across the lips of her customers." This may or may not be so; but in any case "all customers are treated with the same 'stick.'" It is easy to say that such a thing would not be tolerated in England, but when we hear that, notwithstanding the recent fatality from the use of petroleum hair-wash, the demand for that method of removing dirt from the hair is greater than ever, we must confess that we have our doubts as to there being any limit to what some women will put up with for the sake of adding to their personal charms. We cannot speak too strongly against the use of the "rouge stick" as a final touch. Even at home it is a disgusting display of morbid vanity, but when done by a public "stick" it is filthy and dangerous in the extreme.

### The Pauper's Last Possession.

THE growth of our medical schools and the increasing stringency of our medical examinations have tended to increase the demand for "bodies" for purposes of dissection. In fact, but for the increasing use of "pickles" and the extended employment of preservative substances in preparing the "subjects," the proper study of anatomy would become next door to impossible. A little time ago Professor Macalister made an excursion to a large town some distance from Cambridge to beg a few bodies. He met, however, with but little success. The fact is no board of guardians subject to public election cares much to be concerned in this traffic. The "penny-a-liner" is always with us, and we are not altogether surprised at the guardians declining to do anything which might, even as a remote possibility, bring upon them the odium of dealing so harshly with the strangers within their gates. Perhaps, indeed, it occurred to them that they already had enough to answer for. At any rate, they declined the tempting offer, and in doing so expressed the opinion that if the supply of subjects was a matter of such great importance the Government should take it up. We fancy we see a political party risking an election on such a subject! We have, however, received an interesting suggestion from an "Old Nurse," who evidently is in sympathy both with paupers and with education. She suggests that an arrangement should be made by which a friendless pauper might be able to obtain while living the value of his body after death. It is impossible to shut one's eyes to the humorous aspects of this suggestion; but, after all, there is in it a smack of common sense. Why, indeed, should a poor man spend a joyless old age when he possesses such a valuable asset as his own body? Think of the tobacco he could buy and the comforts he might obtain when he had once made his "little arrangement" with the agent of the medical school. Of course, legislation would be required to make it feasible, or we fear that the guardians would bag the profits; but, if such an arrangement were made, surely "subjects" would soon become abundant. The prospect of having a capitalist among them would make public opinion in every infirm ward run strongly in favour of such "deals" in "futures," and, while the pauper would get his due, the student would get his body. Quite a mutual benefit!

### Catching Cold.

IT is not altogether unsatisfactory to people who think that science and common sense should run together, although no doubt discouraging to those who looked on the germ theory of disease as the opening of a sanitary millennium, to find that after all we can "catch cold." The great discovery that most of the febrile diseases from which we suffer are associated with the growth within us of micro-organisms made many people for a time look somewhat sceptically on "catching cold," and we were told that when we felt shivery, and then in a few hours found ourselves sniffling and out of sorts, the chill to which we attributed all the mischief was really the first sign of our being ill. Certain experiments, however, which have recently been made tend to rehabilitate "cold" in its position as a cause of disease, for they have shown that exposure to cold lowers the resistance of the body to infection, and, what is more interesting still, they have made it clear that in regard to various diseases which are known to be caused by micro-organisms, and especially in regard to pneumonia, we may carry the organisms about with us and not suffer, and yet that exposure to cold may at once enable the microbes to take root. Recent demonstrations of the presence of the pneumococcus in the lungs of healthy animals, and the fact that exposing such animals to a thorough chill will bring on pneumonia, is very suggestive, and makes it probable that in many of the ailments which result from "catching cold" a concurrent infection from without is not necessary. The healthier and the cleaner the man, both inside and out, the more, no doubt, will he be able to bear exposure without ill consequences; but for those people whose tissues are already charged with infective micro-organisms, a "mere chill" may evidently set up disease.

